# Clinical outcome and prognostic factors for patients treated within the context of a phase I study: the Royal Marsden Hospital experience

**DOI:** 10.1038/sj.bjc.6604648

**Published:** 2008-09-09

**Authors:** U Tirelli

**Affiliations:** 1 Department of Medical Oncology, National Cancer Institute, Via Franco Gallini 2, Aviano (PN) 33081, Italy

**Sir,**

The evaluation made by Arkenau *et al* (2008) on the impact of progression free and overall survival of 29 phase I trials performed on 212 consecutive patients is noteworthy. However, it is necessary to emphasise that 54 (25%) of these patients are affected by prostate cancer and 37 (68%) of them were not pretreated with chemotherapy. The fact that patients with such urological cancers have had a significant better outcome compared with those with non-urological cancers, *P*=0.001, as reported by the authors, can be justified by the fact that, after phase I trials, these patients not pretreated with chemotherapy were probably treated with Docetaxel regimens, which are known to be efficacious forms of therapy in hormone-resistant prostate cancer. It would be very interesting to compare the outcome of these two groups of patients with prostate cancer, that is, those not pretreated *vs* those pretreated with chemotherapy. In case of no differences in the outcome, it could be stated that patients whose prognosis is poor, despite chemotherapy, could be safely given phase I chemotherapy trials upfront without having been pretreated with any chemotherapy regimen, with obvious advantages for the assessment of new therapeutic drugs.
